# Application of trans-sutural distraction osteogenesis based on an optical surgical navigation system to correct midfacial dysplasia

**DOI:** 10.1038/s41598-022-16013-9

**Published:** 2022-08-01

**Authors:** YuJie Chen, ShanShan Du, ZhiYu Lin, PeiYang Zhang, XinLing Zhang, Yang Bin, JunChen Wang, ZhenMin Zhao

**Affiliations:** 1 Department of Plastic Surgery, Peking University Third Hospital, No. 49 North Garden Road, Haidian District, Beijing, 100191 China; 2 School of Mechanical Engineering and Automation, Beihang University, No. 37 Xueyuan Road, Haidian District, Beijing, 100191 China; 3 The Digital Plastic Surgery, Plastic Surgery Hospital, Chinese Academy of Medical Sciences and Peking Union Medical College, Beijing, 100144 China

**Keywords:** Oral diseases, Characterization and analytical techniques, Imaging techniques

## Abstract

Midfacial hypoplasia is a common maxillofacial deformity in patients with cleft lip and palate, which requires surgical treatment. However, trans-sutural distraction osteogenesis (TSDO) remains some disadvantages, including difficulty in accurate location of surgical path, prolonged operation time, and excess surgical bleeding. This study aimed to evaluate the application of an optical surgical navigation system (OSNS) developed for TSDO. Six consecutive patients with midfacial hypoplasia who required TSDO were included in the study. Preoperatively, a head computed tomography was performed, and the data were imported into Mimics software (version: 20.0.0.691, Materialise Inc, Belgium) to design a three-dimensional simulation of the surgical approach. TSDO was performed with the use of OSNS. The accuracy and results of the procedure were initially evaluated by comparing the preoperative and postoperative periods. The patients included five boys and one girl, with an average age of 10 years; five with postoperative cleft lip and palate, and one without combined cleft lip and palate. The surgical procedure was successful, with a postoperative follow-up of 4–5 months. All patients demonstrated good treatment results without complications. In conclusioin, OSNS-assisted TSDO can noninvasively correct midfacial dysplasia, improve surgical precision, reduce bleeding and obtain better clinical results. OSNS can guide the TSDO safely and effectively.

## Introduction

Midfacial hypoplasia is a common maxillofacial deformity, which is mostly observed in patients with cleft lip and palate^1^. It not only affects the appearance, pronunciation, and breathing, but also hurts patients’ self-esteem and causes serious physical and mental problems^2^. Therefore, the treatment of midfacial hypoplasia has been one of the research hotspots in maxillofacial surgery^3^. In 1990s, traditional orthognathic surgery combined with Le Forts osteotomy was used to correct midfacial hypoplasia. It requires exposure of the maxilla and osteotomy of the bilateral maxilla to achieve the effect of advancement. This method had major disadvantages of large trauma and high technical difficulty^4–6^. In 2005, Liu et al.^7,8^ introduced trans-sutural distraction osteogenesis (TSDO), a minimally invasive and non-osteotomy traction procedure that could effectively correct midfacial dysplasia while avoiding the sequelae associated with osteotomy. Later, Zhao et al.^9–11^ found some disadvantages of TSDO, including difficulty in accurate location of surgical path, prolonged operation time, and excess surgical bleeding. In addition, the long-term follow-up demonstrated that imprecise positioning during TSDO increased the risk of postoperative complications. They found that 8 of 85 patients experienced postoperative complications, three of whom have no traction hooks due to imprecise positioning^12–14^. Thus, although TSDO is the preferred treatment for correction of midfacial dysplasia, there is much room for improvement, and the most critical aspect is to locate the surgical path precisely.

With the rapid development of information technology, many procedures could be more precise with the help of computer-aided surgery (CAS)^15,16^. In CAS, the optical surgical navigation system (OSNS) enables surgeons to observe the surgical path clearly via a display or an augmented reality device. It can effectively improve the accuracy and reduce the difficulty by combining with spatial positioning technology to feedback the position of surgical instruments in real time^17^. Currently, OSNS-assisted positioning of traction hook insertion in TSDO surgery had not been reported yet. The effectiveness and safety of OSNS-assisted TSDO (OAT) require further study. In this paper, we present the surgical model of OAT for the first time and demonstrate its effectiveness and safety by analyzing the medical data of patients retrospectively.

## Results

**Figure 1:**
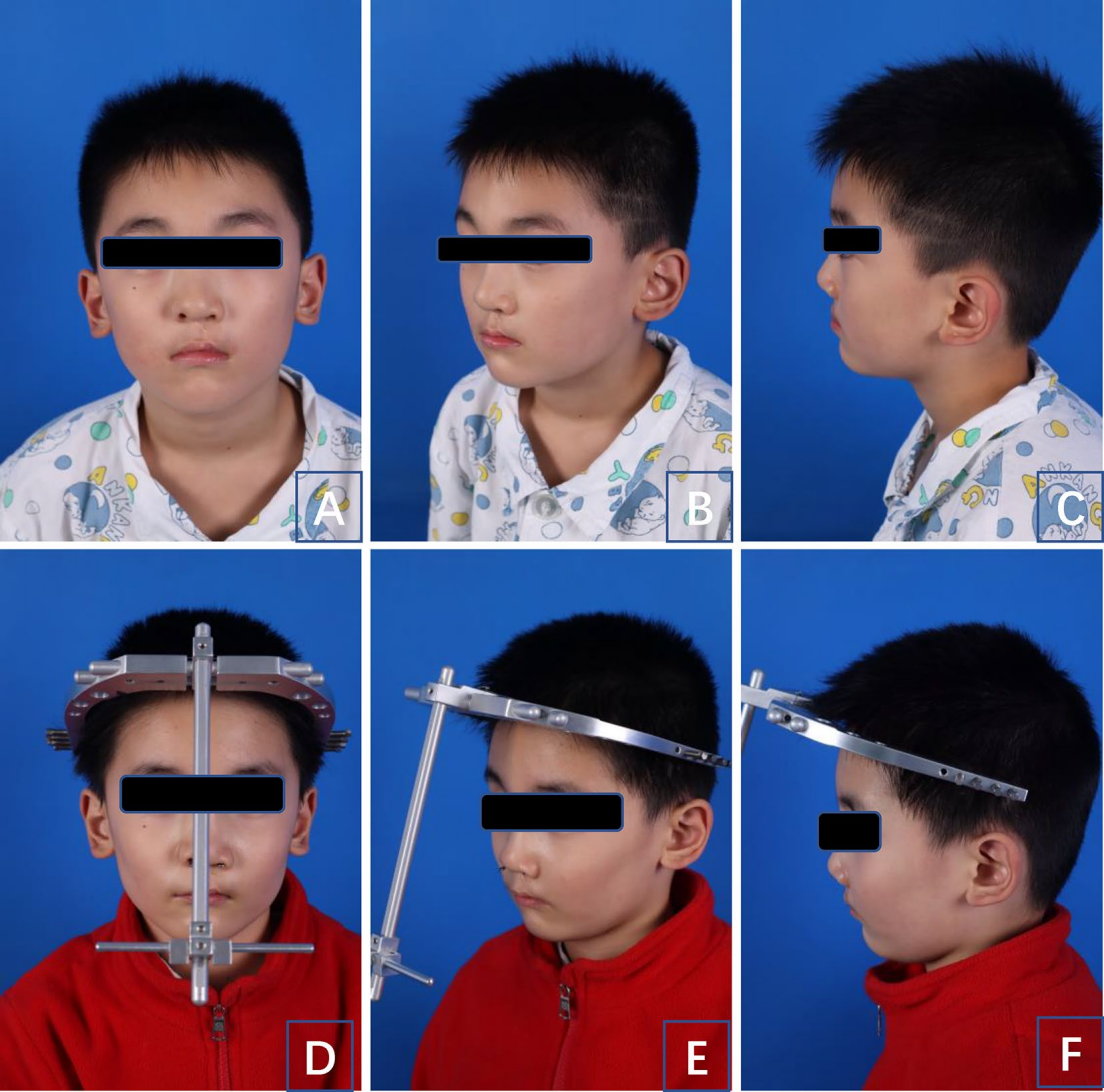
A patient were compared between preoperative and 2-months postoperative photographs (Preoperative: **A** (Front), **B** (Oblique), **C** (Lateral); Postoperative: **D** (Front), **E** (Oblique), **F** (Lateral)).

Six patients were included in this study: five boys and one girl, with a mean age of 10 years. Five patients had midfacial dysplasia combined with cleft lip and palate. All patients underwent OAT. The traction hooks were placed precisely using OSNS intraoperatively, and the procedures went smoothly without any failure of placement. The median intraoperative bleeding was 10 mL, and the average operation time was 6 h. The postoperative follow-up was 4–5 months. All patients recovered well without any complications (Fig. 1). After the traction procedure, the craniofacial imaging data including the sagittal and lateral position showed significant correction of maxillary deformity. The changes in relevant important measurement indexes (SNA, SNB) were statistically significant (P < 0.005).

## Discussion

The treatment of midfacial hypoplasia is of great significance in adolescents. Treatment goals include correction of midfacial deformities; improving appearance, articulation, and breathing disorders; and regaining self-confidence^2^. Traditional orthognathic surgery, osteotomy, and traction surgery often require the use of Le Fort osteotomy techniques, which are traumatic and risky^4^. Hence, they are gradually replaced in clinical practice. Meanwhile, TSDO is a minimally invasive, non-osteotomy procedure that has become the preferred choice for children with midfacial hypoplasia^9,10^. However, a minimally invasive incision is often required to expose the maxilla and define the surgical path for observation and protection of the adjacent roots and nerves in the maxilla.

Artificial intelligence (AI) is used to assist operations and achieve better surgical^18–20^. It has also been adopted in craniofacial surgeries, such as intelligent analysis and intelligent navigation. However, due to operative inconvenience caused by the head positioning device, AI hasn’t been used in TSDO. Currently, intelligent navigation is a rising technology in surgical assistance. OSNS has gained widespread interest owing to its enhanced ability to visualize surgery-related dynamics^17^. Currently, reports on the application of OSNS in TSDO are lacking. It will be an extraordinary innovation to combine TSDO and OSNS.

In this study, we presented the application of an OSNS developed by our center in TSDO. TSDO was performed with the assistance of OSNS in six patients through the real-time dynamic guidance of OSNS. The procedure was successful in all patients, avoiding incisions of the mucosa and nasal base in the oral cavity and realizing true “minimal invasiveness”. The average intraoperative bleeding volume was 7.5 ml (Mean ± SD, 7.5 ± 2.74), compared to 13.8 mL (Mean ± SD, 13.8 ± 3.77) during conventional TSDO in our center^10,12^. This showed the trend that OAT could reduce the bleeding volume of operation. Moreover, OAT helped to maximize the protection of patients’ tooth roots and nerves, greatly reduced the difficulty of traction hook placement, and significantly reduced the occurrence of postoperative complications while laying the foundation for obtaining stable traction results later. In terms of operation time, OAT is slightly shorter than traditional TSDO (6 ± 1.14 vs. 6.5 ± 1.02), but the time advantage was not significant. It may be related to the fact that OAT is a novel method and the surgeons were not able to operate it skillfully yet. It is reasonable to believe that as the application of OSNS becomes more popular, the operation time will be further reduced.

Here are some of the advantages of using OAT. First, OSNS is generally adopted to the process of TSDO without too much extra learning cost. Second, via modeling and preoperative visualized images, it can guide the surgical path in real time, which is of great help for precise medical treatment. Third, it allows dynamic observation of the situation related to the roots in maxilla, which minimizes the damage to roots and teeth. Fourth, the OSNS does not possess frontal fixed signal receiver so that it greatly increases the area of operation through the positioning kit out of the braces. Lastly, the 3D Mimics 20.0 software can be used to assess the clinical effect before and after traction and define the traction site to provide more precise guidance for subsequent research^17,21^.

With OSNS, the operative design can be tailored, and the most suitable surgical path can be chosen for different individuals. For surgeons, this combination removes the difficulty in observing the surgical path dynamically without incision in traditional TSDO and further shortens the learning curve^22,23^. For patients, accurate operation and precise placement of the traction hook can achieve better surgical results with minimal trauma and less complications.

OAT can aid surgeons in performing surgery more precisely, which can effectively improve the efficiency and safety of surgery and reduce operative trauma. However, this study had some limitations. First, the surgery time in the early stage of learning OAT may be slightly longer, although it could be shortened with proficiency in operation. Second, the sample size was small because OAT is just recently applied for clinical practice. Nevertheless, we intend to expand the sample size and combine it with prospective longitudinal clinical studies in the future to obtain more effective results and explore the advantages of OAT further.

In conclusion, OAT can correct midfacial dysplasia effectively and safely. It is minimally invasive, improves surgical precision, and reduces bleeding. OAT also accelerates the promotion and application of TSDO-related surgery, which is another milestone for midfacial hypoplasia correction.

## Methods

### General information

**Figure 2:**
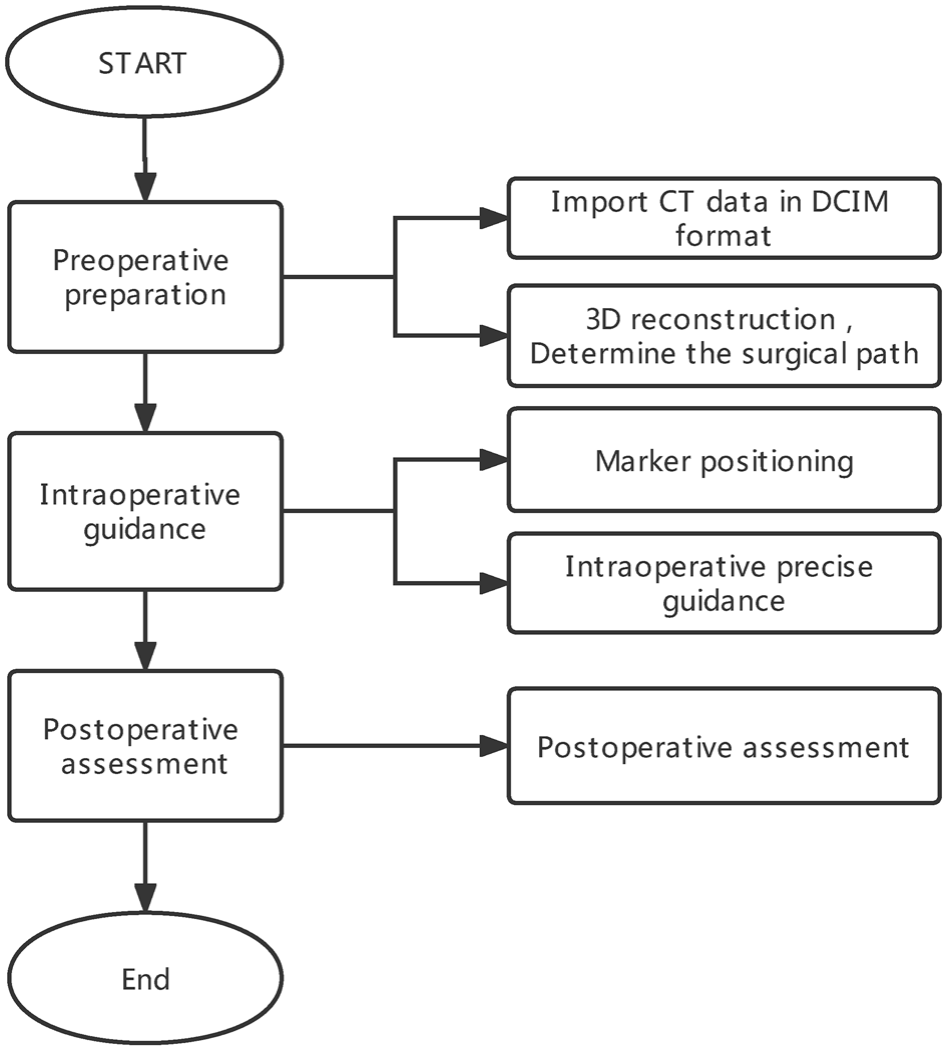
The workflow of the whole process.

Six patients with midfacial hypoplasia who were admitted to the Department of Plastic Surgery, Peking University Third Hospital from July 2021 to August 2021 were enrolled in this study. General indicators, such as patient’s age, sex, operation method, and bleeding volume were collected. The patient and demographic characteristics of the patients are summarized in Table 1. All medical protocols in this study adhered to the Declaration of Helsinki. This study was approved by the Institutional Review Board of Peking University Third Hospital (approval number: M2021645). All methods were performed in accordance with the relevant guidelines and regulations of our hospital. Informed consent to release information and images from online open-access publications was obtained from all participants. Figure 2 shows the workflow applied by surgeons and mechanical engineers.

**Table 1:** Characteristics of the 6 patients included in the study.

Patient	Age (years old)	Sex	Disease	Cleft lip and palate	Complication	Time (hours)	Bleeding (ml)	Follow-up period(months)
1	7	M	Maxillary retrusion	Y	N	5.5	10	5
2	10	M	Maxillary retrusion	Y	N	7	5	5
3	9	M	Maxillary retrusion	Y	N	7	5	4
4	12	M	Maxillary retrusion	Y	N	6.5	10	4
5	9	M	Maxillary retrusion	Y	N	6	10	5
6	13	F	Maxillary retrusion	N	N	4	5	4

*M* male, *F* female, *Y* yes, *N* none.

### Details of the optical surgical navigation system

**Figure 3:**
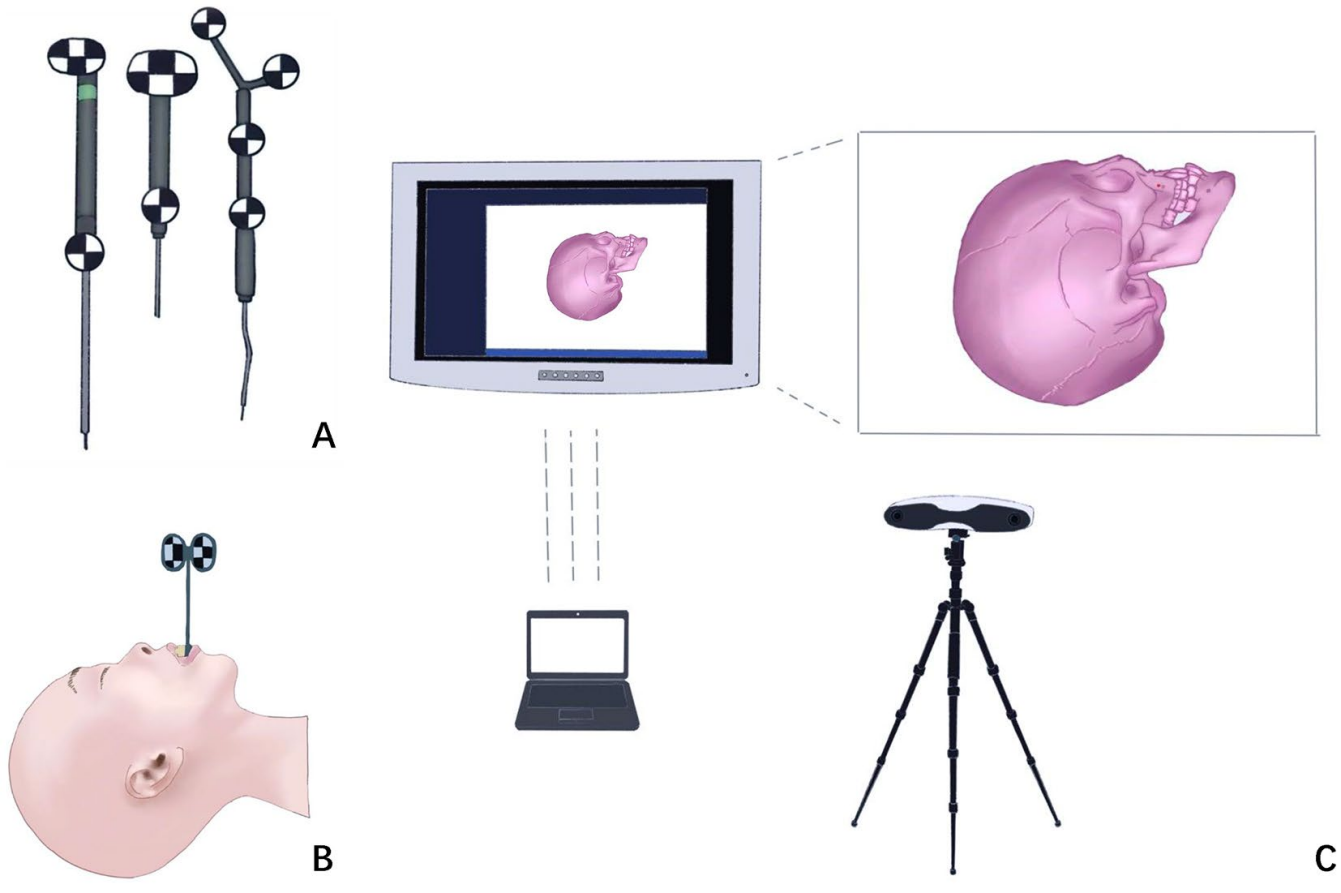
Compositions of OSNS system.

The OSNS used in this study mainly consists of a real-time detection device (Fig. 3A), a marker localization device (Fig. 3B), a bridge set, and a binocular navigator (GS3-U3-41C6M-C, Point Grey), a display and a computer (Fig. 3C). The binocular navigator uses a computar prime lens with a focal length of 12 mm, and the baseline distance of the binocular camera is set to 300 mm (suitable for the medical field of view), the image acquisition frequency is 30 ~ 40 Hz, and the image resolution is 2048 × 2048.

The binocular navigator, which was built from two CCD cameras, is similar to an "eye" that acquires images in real time and transmits them to the PC for processing (the PC is similar to the brain, which performs the algorithmic analysis). X-shaped feature corner points detected by binocular navigation (as shown in the Fig. 3C) calculate the parallax of the two matching feature points. It later calculates the 3D coordinate values of the feature points under the camera coordinate system according to the triangulation principle. Lastly, it completes the line alignment of the two images through aberration correction and stereo correction and complete the stereo matching. The local coordinate system was established using principal component analysis before the operation, and then the coordinate values of these feature points in this coordinate system are recorded and stored as templates. During the operation, the template matching was performed based on the detected feature points. The position and pose of the successfully matched visual marker in the camera coordinate system were then calculated.

To ensure the uniqueness and stability of the local coordinate system of visual markers, the OSNS used in this study adopts the Fast Triangle Screening (FTS) algorithm, which optimizes the process of template matching and saves a lot of calculation time. The three feature points form a triangle, which is the triangle with the largest area among the four points. The triangle was used as the standard (Fig. 3A). When tracking binocular vision, the main triangle will be matched by the area feature. If the area error is within the allowable range, it means that the combination of the three points selected is correct, but the order of the three points is not necessarily correct. It is necessary to further judge whether the arrangement order is correct through the characteristics of the maximum angle and the minimum angle. Such a screening method will greatly save the amount of computation, improve the calculation efficiency of the system, and ensure the real-time performance of the vision of the surgical robot.

Binocular stereo vision navigation is used in clinical applications, where the surgeon relies on the guidance of a visual image model to perform the surgery. The relative position of the surgical probe to the patient is acquired directly from the screen image (Fig. 3C).

### Procedure

#### Step 1: Pre-operative preparation

Preoperatively, a dual-source computed tomography (CT) scanner (SOMATOM Definition Flash 128 Rao, Siemens, Germany) was used to perform a spiral scan of the patient's whole skull with a layer thickness of 0.5 mm. The CT data were imported into the Mimics 20.0 software (version:20.0.0.691, Materialise Inc, Belgium) in DICOM format for craniomaxillofacial 3D reconstruction. Model 3D printing was performed using a Stratasys, Objet 500 Connex 3 3D printer. The pre-designed surgical design, which represents the traction hook placement perforation points, was reflected on the printed model.

#### Step 2: Components and working principle

**Figure 4:**
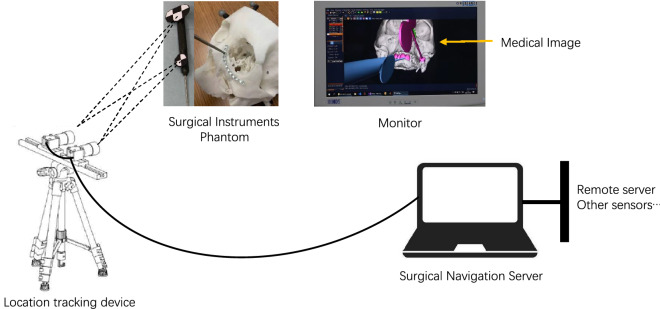
Working principle of optical navigation system. Identification of the probe and the Marker on the braces by the binocular lens for positioning on the model, while the probe and the reconstructed model of the skull are presented in real time on the monitor.

The OSNS was based on the following components: binocular navigators, monitors, marker positioning devices, braces, bridging sleeves, and real-time probe devices. The principle was to use intraoperative real-time tracking and positioning technology to calculate the position of the surgical operation target point in relation to the surgical instruments or probes in real time. Next, the data were rendered and displayed in real time on the monitor to provide accurate surgical guidance to the surgeon. The system hardware includes several parts, such as a binocular lens, computer host, and monitor to identify and position the marker. The alignment simulation was performed on the 3D printed model through an OSNS to test the alignment accuracy. The cranial model was recognized by the binocular lens through the marker on the brace struts and on the surgical instruments. The image was then transmitted to the computer software in real time, which facilitates the simultaneous observation of the operation through the monitor. The alignment accuracy error was < 2 mm by more than five simulations preoperatively (Fig. 4).

#### Step 3: OSNS for intraoperative guidance

**Figure 5:**
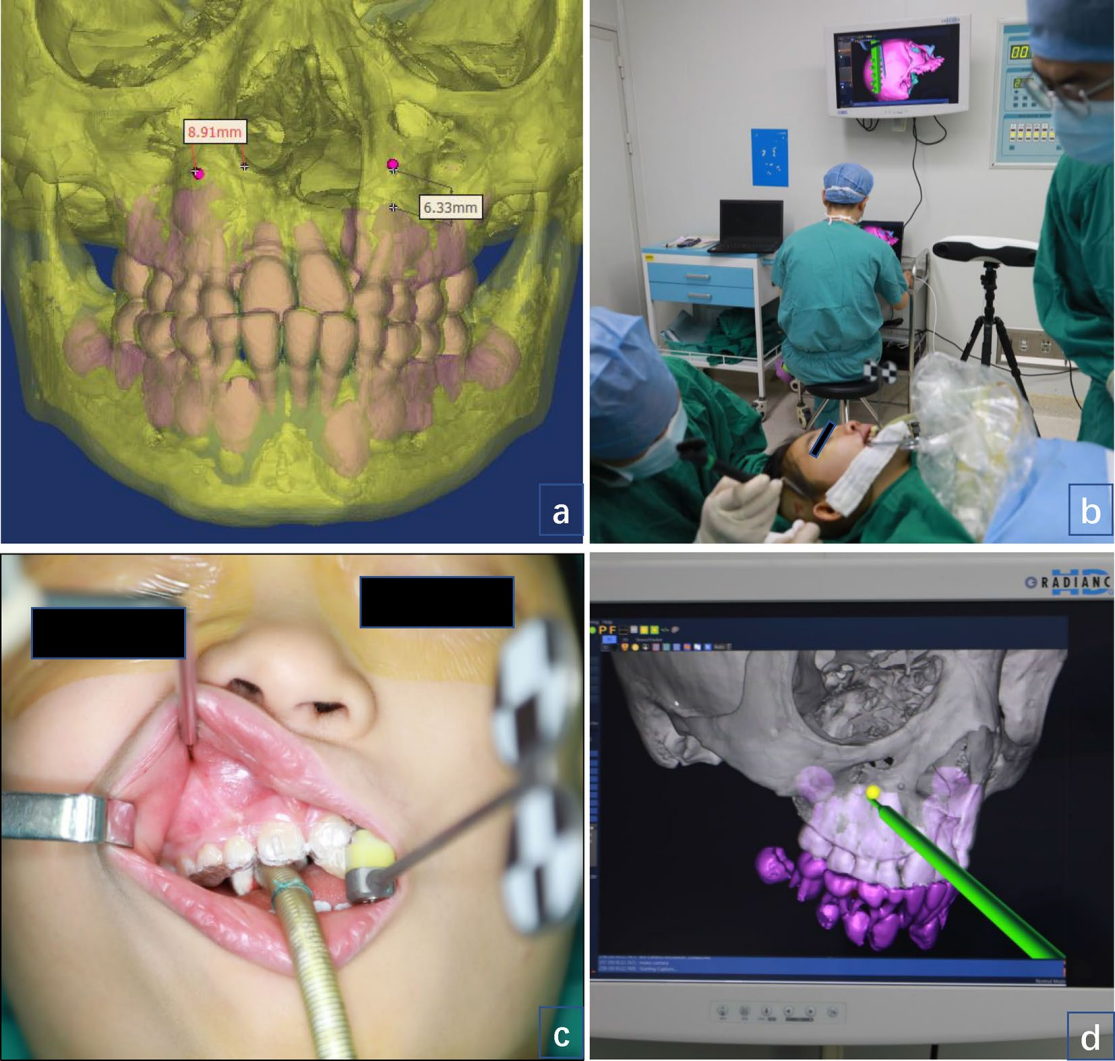
Illustrates the real-time process of guiding the surgery, dynamically based on this navigation system. (**a**) Preoperative design of traction hook placement points; (**b**) the position of the fixed screw punching can be reflected in the scalp projection point in real time through the OSNS intraoperatively; (**c**,**d**) With the assistance of the navigation system, the surgeon could observe the real-time location of the probe through the display screen without cutting the upper lip mucosa, separating the tissues to expose the maxilla.

Figure 5 illustrates the real-time process of guiding the surgery based on the OSNS.

During the operation, this navigation system was used to locate the maxilla at the outer edge of the bony pyriform foramen on both sides. The surgical access was clearly defined at 6 mm above the root of the cuspid and approximately 1 cm from the lateral edge of the pyriform foramen. With the assistance of the OSNS, the surgeon could observe the real-time location of the probe through the display screen. This greatly reduces soft tissue damage while protecting the roots of the teeth. For the placement of the cephalic frame, it can be positioned by precise measurement preoperatively, and the position of the fixed screw punching can be reflected in the scalp projection point in real time through the OSNS intraoperatively to avoid visual errors.

#### Step 4: Evaluating the safety and effectiveness of the OSNS

**Figure 6:**
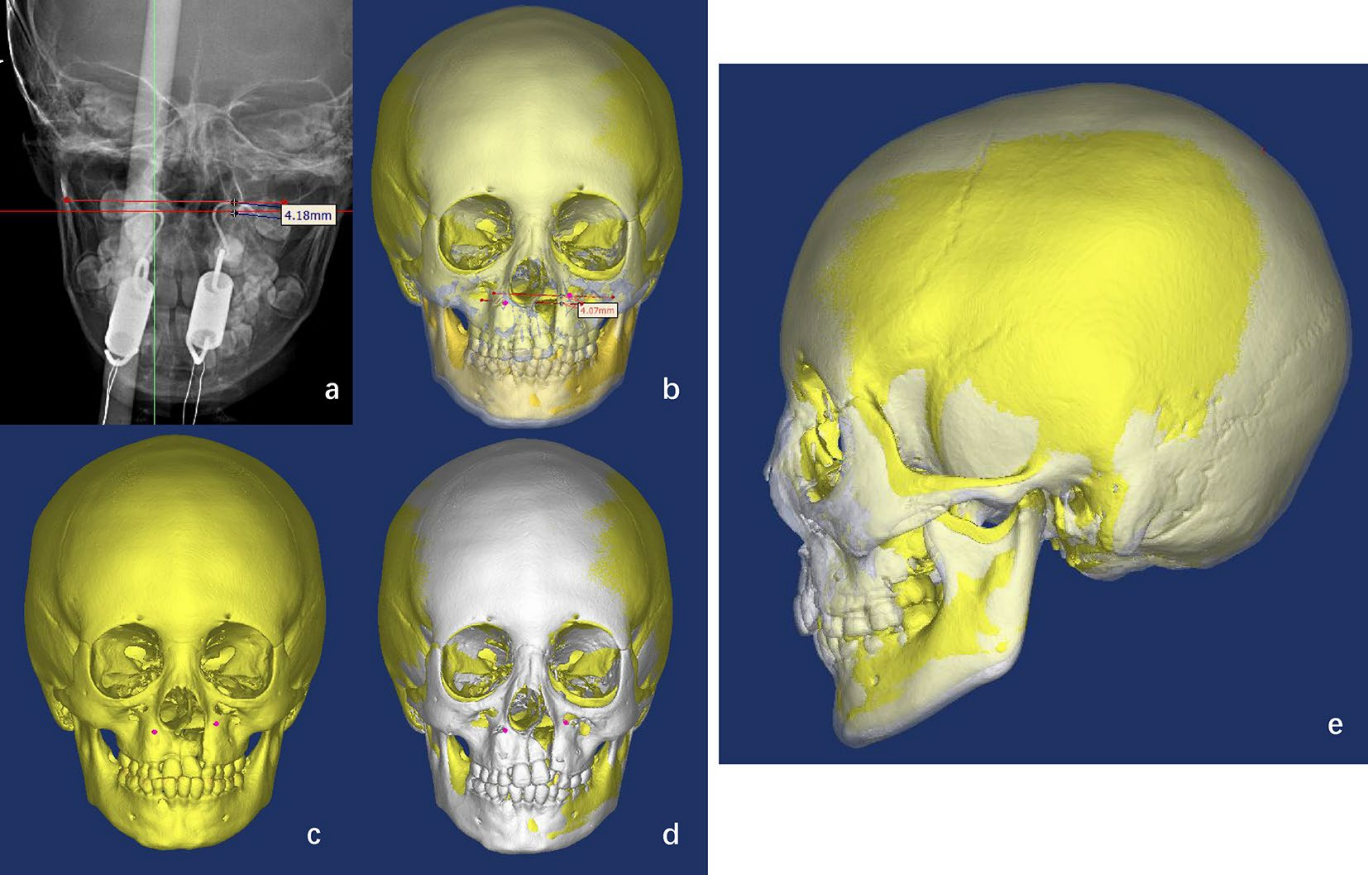
Evaluating the safety and effectiveness of the OSNS. (**a**) A cranial X-ray was performed to clarify the position of the traction hook; (**b**–**d**) The preoperative 3D reconstructed image was used as the base coordinate system, and the 3D cranial model was reconstructed after treatment (after removal of the traction hook), fitted to the base coordinate system to measure the changes in the morphological position of the patient’s maxilla; (**e**) The morphological position of the patient’s maxilla before and after treatment.

Postoperatively, a cranial X-ray was performed to clarify the position of the traction hook and calculate the bleeding volume. After traction, the head frame was removed. A cranial CT was reviewed to evaluate the traction effect. The preoperative 3D reconstructed image was used as the base coordinate system, and the 3D cranial model was reconstructed after treatment (after removal of the traction hook). The model was fitted to the base coordinate system to measure the changes in the morphological position of the patient’s maxilla before and after treatment and to verify the relationship between the desired traction hook placement points and the actual postoperative position points to verify the accuracy of the optical navigation system. The coordinates of the bilateral traction hook placement points were measured in 3D space before and after treatment in two patients (Fig. 6 and Table 2).

**Table 2:** Three-dimensional spatial position and SNA and SNB angles of the preoperative and postoperative traction hook placement points.

Patient	Operation	X (transverse)		Y (sagittal)		Z (coronal)		SNA	SNB
		A (left)	B (right)	A (left)	B (right)	A (left)	B (right)		
1	Pre-	67.5	64.5	132.7	102.7	189.4	196,2	69°	71°
	Post-	66.5	63.5	133.8	102.2	191.8	199.1	76°	71°
2	Pre-	180.5	184	145.1	98.7	286.7	191.9	86°	80°
	Post-	179.5	183.5	145.6	98.3	193.8	197.1	97°	82°
3	Pre-	95.5	93.5	124.1	83.6	161.8	164.3	87°	77°
	Post-	95	93	125	84	167.7	169	95°	78°
4	Pre-	83	81	137	95	174	178	85°	86°
	Post-	83	80	138	95.3	178.2	183	93°	86°
5	Pre-	98	95	145.1	108.7	158.7	159.9	83°	85°
	Post-	98	94	144.8	107.7	164.6	164.2	92°	86°
6	Pre-	84	84.5	158.4	122	192.6	189.1	84°	83°
	Post-	83.5	84	158.6	122.3	197.8	194.1	90°	84°
P								< 0.001	0.004

It can be seen that through the maxillary traction surgery, there is little change in the position of the bilateral traction points to the transverse axis and sagittal axis, but there is a significant difference to the coronal axis, indicating a significant anteriority of the maxilla. By preoperative and postoperative SNA and SNB angle measurements, it can also be seen that there is a significant anteriority of the maxilla and no significant change in the mandible, with significant improvement in the Underbite.

### Brief steps of the operation

The patient was placed in a supine position, and braces were fitted. After administration of anesthesia, the nasal and oral cavities were routinely disinfected and rinsed with a diluted iodophor. The preoperatively designed surgical access was initially defined using the OSNS. Along the mark toward the pear-shaped foramen in the nasal aperture, mechanical perforation was performed using a K-wire to avoid the root of the tooth. After successful perforation, retraction hooks were placed through the nostrils. The contralateral side was treated similarly. Using the OSNS to locate the precise site of cephalic brace placement on both sides of the temporal top, an incision of approximately 4–5 mm in length was made along the direction of the superficial temporal artery terminal alignment using a Melan marker. The scalp was then incised. Vascular forceps were used to bluntly separate the subcutaneous tissue to the periosteum, and the cephalic brace was fixed using titanium nails. The vertical and horizontal rods of the traction brace were installed.

## Data Availability

The datasets developed and analyzed during this study are available from the corresponding author on reasonable request. All datasets generated for this study are included in the article, Patient names are inconvenient to provide because of the need to protect clinical patient privacy.
